# Defects in fatty acid amide hydrolase 2 in a male with neurologic and psychiatric symptoms

**DOI:** 10.1186/s13023-015-0248-3

**Published:** 2015-03-28

**Authors:** Sandra Sirrs, Clara DM van Karnebeek, Xiaoxue Peng, Casper Shyr, Maja Tarailo-Graovac, Rupasri Mandal, Daniel Testa, Devin Dubin, Gregory Carbonetti, Steven E Glynn, Bryan Sayson, Wendy P Robinson, Beomsoo Han, David Wishart, Colin J Ross, Wyeth W Wasserman, Trevor A Hurwitz, Graham Sinclair, Martin Kaczocha

**Affiliations:** Departments of Medicine, University of British Columbia, Vancouver, Canada; Departments of Pediatrics, University of British Columbia, Vancouver, Canada; Centre for Molecular Medicine and Therapeutics, Child and Family Research Institute, University of British Columbia, Vancouver, Canada; Treatable Intellectual Disability Endeavour in British Columbia (TIDE-BC), Vancouver, Canada; Department of Anesthesiology, Stony Brook University, Stony Brook, NY 11794-8480 USA; Departments of Biological and Computing Sciences, University of Alberta, Edmonton, T6G 2E8 Canada; Half Hollow Hills High School, Dix Hills, NY 11746 USA; Graduate Program in Molecular and Cellular Biology, Stony Brook University, Stony Brook, NY 11794 USA; Department of Biochemistry and Cell Biology, Stony Brook University, Stony Brook, NY 11794-5215 USA; Department of Medical Genetics, University of British Columbia, Vancouver, Canada; Psychiatry, University of British Columbia, Vancouver, Canada; Department of Pathology and Laboratory Medicine, University of British Columbia, Vancouver, Canada; Division of Biochemical Diseases, Rm K3-201, Department of Pediatrics, B.C. Children’s Hospital, Centre for Molecular Medicine & Therapeutics, University of British Columbia, 4480 Oak Street, Vancouver, B.C. V6H 3V4 Canada

**Keywords:** Fatty acid amide hydrolase 2, FAAH1, FAAH2, Anandamide, Endocannabinoids, Intellectual developmental disability, Ataxia, Anxiety, Psychiatric diseases

## Abstract

**Background:**

Fatty acid amide hydrolase 2 (FAAH2) is a hydrolase that mediates the degradation of endocannabinoids in man. Alterations in the endocannabinoid system are associated with a wide variety of neurologic and psychiatric conditions, but the phenotype and biochemical characterization of patients with genetic defects of FAAH2 activity have not previously been described. We report a male with autistic features with an onset before the age of 2 years who subsequently developed additional features including anxiety, pseudoseizures, ataxia, supranuclear gaze palsy, and isolated learning disabilities but was otherwise cognitively intact as an adult.

**Methods and results:**

Whole exome sequencing identified a rare missense mutation in *FAAH2,* hg19: g.57475100G > T (c.1372G > T) resulting in an amino acid change (p.Ala458Ser), which was Sanger confirmed as maternally inherited and absent in his healthy brother. Alterations in lipid metabolism with abnormalities of the whole blood acyl carnitine profile were found. Biochemical and molecular modeling studies confirmed that the p.Ala458Ser mutation results in partial inactivation of FAAH2. Studies in patient derived fibroblasts confirmed a defect in FAAH2 activity resulting in altered levels of endocannabinoid metabolites.

**Conclusions:**

We propose that genetic alterations in FAAH2 activity contribute to neurologic and psychiatric disorders in humans.

**Electronic supplementary material:**

The online version of this article (doi:10.1186/s13023-015-0248-3) contains supplementary material, which is available to authorized users.

## Background

The endocannabinoid system (ECS) is a complex neural signaling system that is implicated in a wide variety of normal and pathological neurological processes. Although activation of the ECS has been proposed to have neuroprotective effects in a number of disease states, abnormal activation of the ECS can also be neurotoxic [1] and alterations in the ECS have been implicated in a number of neurological diseases including Parkinson’s disease [2], Huntington’s disease [3], multiple sclerosis [4] and Fragile X syndrome [5]. Alterations in endocannabinoid signaling have also been implicated in a wide variety of psychiatric conditions including anxiety, depression, schizophrenia [6-14] and autistic spectrum disorders [15,16].

The endocannabinoids anandamide (AEA) and 2-arachidonoylglycerol (2-AG) are degraded by a series of four hydrolases in man [17]. Alterations in endocannabinoid activity can occur through altered receptor binding [8], alterations in metabolic enzymes [18] or downstream pathways [13]. Fatty acid amide hydrolase (*FAAH1*; [MIM 606581]) is the principal enzyme that hydrolyzes AEA in mammals [18]. Missense variants in *FAAH1* have been associated with schizophrenia in genome-wide associated studies [19] and homozygosity for a common polymorphism in *FAAH1* reduces functional activity of the enzyme and is associated with problem drug use [20] which is a model for psychiatric disease. However, recently a second enzyme (*FAAH2* [MIM 300654]), present in man but not rodents, was identified and shown to mediate endocannabinoid degradation [21,22]. Inhibition of FAAH1 or FAAH2 would be expected to increase levels of endocannabinoids available for receptor binding. The ECS is implicated in neural development [10] and overactivation of the ECS during pregnancy has been associated with growth and neurocognitive deficits in human offspring [23-25]. Thus it is conceivable that mutations that affect FAAH2 enzyme activity could result in a neurologic or psychiatric phenotype.

The *FAAH2* gene resides on the X chromosome in man and has been identified in recent genome wide association studies as a possible candidate gene for X-linked intellectual disability [26] and autism spectrum disorders [27]. Here, we present a novel case where a male patient with neurologic and psychiatric symptoms was shown to harbor a distinct missense variant in the *FAAH2* gene. Using a variety of techniques, we provide evidence that this mutation compromises FAAH2 activity and propose that this alteration in endocannabinoid signaling may be the cause of the phenotype observed in this patient.

## Methods

### Ethical issues

This study was initiated as part of the Treatable Intellectual Disability Endeavor in British Columbia. Informed consent was obtained from the individuals involved in this study and approved by the ethics committees of the University of British Columbia (Vancouver, Canada).

### Whole exome sequencing

Genomic DNA was isolated from the peripheral blood of the patient, unaffected brother, as well as parents using standard techniques. Whole exome sequencing was performed for all four family members using the Ion AmpliSeq™ Exome Kit and Ion Proton™ System from Life Technologies (Next Generation Sequencing Services, UBC, Vancouver, Canada). An in-house designed bioinformatics pipeline [28] was used to align the reads to the human reference genome version hg19 and to identify and assess rare variants for their potential to disrupt protein function. The average coverage was 100X. Rare variants were identified based on a comparison against alleleic frequencies from dbSNPv138, Exome Variant Server and an in-house database of more than 260 exomes and genomes using minor allele frequency (MAF) as <1% as the threshold. The remaining variants were subsequently screened under a series of genetic models described in the text. We have submitted the *FAAH2* missense variant to the LSDB gene variant database (http://grenada.lumc.nl/LOVD2/MR/variants?action=search_unique&select_db=FAAH2).

### Cloning and transfections

Human FAAH1 (NM_001441) and FAAH2 (NM_174912) cDNAs were subcloned into pcDNA4 expression vectors. A FLAG epitope tag was inserted in the C-terminus of FAAH2. Site-directed mutagenesis was performed using Quikchange. All constructs were verified by DNA sequencing. Human 293T cells and primary fibroblasts were cultured in DMEM supplemented with 10% fetal bovine serum, 100 U/ml penicillin/streptomycin, and 2 mM L-glutamine. Transfections were performed using the GenJet Plus transfection reagent (SignaGen, Rockville, MD) according to the manufacturer’s instructions.

### Western blotting

Western blot experiments were performed exactly as described [29]. Blots were probed mouse anti-FAAH1 (1:1000, Abcam #Ab54615), mouse anti-GAPDH (1:5000, Abcam #Ab8245), mouse anti-FLAG (1:1000, Sigma #F1804), or rabbit anti-FAAH2 (1:200, Abcam #Ab103724) antibodies. The blots were then incubated with the respective goat anti-mouse or goat anti-rabbit IgG HRP-conjugated antibodies (Life Technologies), developed using the Immun-star HRP substrate (Bio-Rad), and scanned using a C-DiGiT scanner (Li-COR).

### Enzyme assays

Enzyme assays using [^14^C]AEA or [^14^C]PEA as substrates were performed exactly as described [22].

### Endocannabinoid quantification in fibroblasts

Endocannabinoid levels were quantified exactly as described [30]. Briefly, two million affected and unaffected fibroblasts were harvested and the lipids extracted by addition of 2:1:1 chloroform:methanol:Tris containing the appropriate deuterated standards, followed by two rounds of centrifugation at 3000 g. The chloroform phase was collected, dried, and the lipids resuspended in 2:1 chloroform:methanol and quantified on a Thermo TSQ Quantum Access Triple Quadropole mass spectrometer.

### Structure modeling and analysis

A FAAH2 model structure was produced by threading the sequence of wild-type human FAAH2 onto a humanized rat FAAH1 template structure (PDB ID:2WAP) in Modeller 9.13 [31]. The p.Ala458Ser mutation was modeled in Coot [32] with a rotamer selected to minimize steric clashes as determined by Molprobity [33]. Atoms within 3.5 Å and capable of forming hydrogen bonds with the hydroxyl group of p.Ser458 were identified in Coot.

### Lipidomic assessment using combined direct flow injection and LC-MS/MS compound identification and quantification

We have applied a targeted quantitative metabolomics approach to analyze the serum samples using a combination of direct injection mass spectrometry (Absolute*IDQ*™ Kit) with a reverse-phase LC-MS/MS Kit. The Kit is a commercially available assay from BIOCRATES Life Sciences AG (Austria). This kit, in combination with an ABI 4000 Q-Trap (Applied Biosystems/MDS Sciex) mass spectrometer, can be used for the targeted identification and quantification of up to 180 different endogenous metabolites including amino acids, acylcarnitines, biogenic amines, glycerophospholipids, sphingolipids and sugars. The method used combines the derivatization and extraction of analytes, and the selective mass-spectrometric detection using multiple reaction monitoring (MRM) pairs. Isotope-labeled internal standards and other internal standards are integrated in Kit plate filter for metabolite quantification. The Absolute*IDQ* kit contains a 96 deep-well plate with a filter plate attached with sealing tape, and reagents and solvents used to prepare the plate assay. First 14 wells in the Kit were used for one blank, three zero samples, seven standards and three quality control samples provided with each Kit. All the serum samples were analyzed with the AbsoluteIDQ kit using the protocol described in the AbsoluteIDQ user manual. Briefly, serum samples were thawed on ice and were vortexed and centrifuged at 13,000 × g. 10 μL of each serum sample was loaded onto the center of the filter on the upper 96-well kit plate and dried in a stream of nitrogen. Subsequently, 20 μL of a 5% solution of phenyl-isothiocyanate was added for derivatization. After incubation, the filter spots were dried again using an evaporator. Extraction of the metabolites was then achieved by adding 300 μL methanol containing 5 mM ammonium acetate. The extracts were obtained by centrifugation into the lower 96-deep well plate, followed by a dilution step with kit MS running solvent. Mass spectrometric analysis was performed on an API4000 Qtrap® tandem mass spectrometry instrument (Applied Biosystems/MDS Analytical Technologies, Foster City, CA) equipped with a solvent delivery system. The samples were delivered to the mass spectrometer by a LC method followed by a direct injection (DI) method. The Biocrates MetIQ software was used to control the entire assay workflow, from sample registration to automated calculation of metabolite concentrations to the export of data into other data analysis programs. A targeted profiling scheme was used to quantitatively screen for known small molecule metabolites using multiple reaction monitoring, neutral loss and precursor ion scans.

## Results

**Figure 1:**
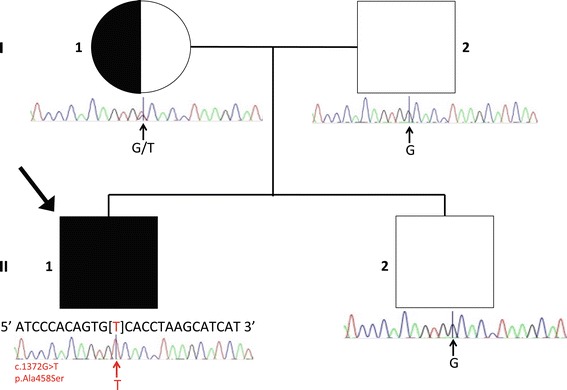
**Pedigree and Sanger sequencing of a patient with** ***FAAH2*** **mutation.** Pedigree information for our patient and Sanger sequencing results are shown. The pedigree is consistent with X-linked recessive inheritance as the mother is clinically unaffected.

Our patient (Subject II-1 in Figure 1) is a 25 year old male who was born after an uncomplicated pregnancy from healthy non-consanguinous Caucasian parents. He has a healthy brother and extended family history is negative for similarly affected children or neuropsychiatric disorders. He presented as a neonate with hypotonia, feeding difficulties and central apneic episodes associated with an abnormal EEG which demonstrated an overabundance of multifocal sharp waves against a discontinuous asynchronous background that was abnormal for age. He had delayed motor and language milestones and as a child was noted to have ocular apraxia with a moderate to severe dysarthria made worse by a mechanically based limitation in jaw opening.

He developed autistic features by the age of 3 years and was diagnosed with an anxiety disorder at the age of 7 which responded to selective serotonin reuptake inhibitors. He remained stable until the age of 22 years when he presented with recurrent psychogenic seizures characterized by episodic atonic collapses heralded by a fluttering of his eyes and then becoming unable to speak or move but retaining full awareness of his environment. These episodes would last seconds up to 30 minutes and occurred once to several times per day. Psychosocial functioning until age 22 years had been relatively stable. He completed school with the help of a scribe because of problems with hand co-ordination. At the time of presentation he was enrolled in university and was studying to obtain a joint degree in accounting and financing with similar assistance.

His neurological examination at the age of 25 years demonstrated preserved neurocognitive abilities, severe dysarthria, a supranuclear vertical gaze palsy, left facial hypoplasia, limited jaw opening (despite MRI confirmed normal temporomandibular joint anatomy showing only very limited anterior translation of the mandibular head on mouth opening), limited lateral movement of his tongue but no associated tongue weakness, bilaterally reduced fine motor movements, a mild left spastic gait and a bilateral action tremor. Mental status examination was significant for a stereotypy characterized by the placement of his fisted hands against his head and mostly surfacing during periods of emotional arousal. His mood was initially reported as euthymic but subsequently he became aware of an underlying depression, which was intermittently associated with suicidal ideation and anxiety often preceding his psychogenic seizures.

Brain magnetic resonance imaging (MRI) at the age of 10 years showed delayed myelination. Repeat MRI at the age of 22 years was unremarkable other than a prominent cisterna magna.

Over time it became clear that his psychogenic seizures represented conversion reactions provoked by severe anxiety from a decompensation into a major depressive illness. As his illness unfolded, his episodes became modified by self-punishing behaviors, which were manifestations of emotional conflicts. He had a partial response to the combination of sertraline, bupropion and quetiapine combined with psychotherapy.

Biochemical work up was negative except for an abnormal whole blood acylcarnitine profile which showed persistent 10-fold elevations in medium chain species with lesser elevations in short and long chain species. Although the patient did not have clinical features to suggest other disorders which could cause this profile including multiple acylCoA dehydrogenase deficiency (*MADD* [MIM 231680]), and defects in riboflavin metabolism, these were excluded through single gene sequencing. The patient did not have a pattern of clinical deterioration to suggest Niemann-Pick Disease type C (*NPC1* [MIM 257220] and *NPC2* [607625]) but, given the gaze palsy, defects in *NPC1* and *NPC2* were excluded through single gene sequencing.

Whole exome sequencing was performed as described in the Methods section. We analyzed the variants predicted to be functional (missense, nonsense and frameshift changes, as well as in-frame deletions and splice-site effects) under a series of inheritance models. We only focused on the variants not present in the clinically unaffected brother. We identified six hemizygous candidate genes (*FAAH2*, *KIAA1210*, *AKAP14*, *TXLNG*, *ZMYM3* and *NRK*), five compound heterozygous candidate genes (*MUC16*, *CFTR*, *TTN*, *KEAP1* and *HIVEP2*) and one gene (*MYO1H*) affected by a *de novo* mutation (see Additional file 1: Table S1). All 12 genes were evaluated to see if any of the candidates could be associated with the neuropsychiatric phenotype and the abnormal acylcarnitine profile, which was present in this patient. The hemizygous variant located on the X-chromosome affecting *FAAH2* (hg19: g.57475100G > T) was considered a very strong candidate in that it harbored a missense mutation c.1372G > T (p.Ala458Ser), FAAH2 is known to play a role in lipid metabolism [21] and alterations in endocannabinoid metabolism have been documented in many different psychiatric disorders [6]. None of the other candidate genes are known to play a role in lipid metabolism and psychiatric diseases (Additional file 1: Table S1). This variant was confirmed by Sanger sequencing (Figure 1) as present in the index case and mother (who had a normal acylcarnitine profile) and was absent in the father and unaffected brother. The variant is reported in dbSNP (version 138) as heterozygous (rs147173444), not hemi/homozygous, with a low frequency 0.00022, but unreported in NHLBI ESP and our in-house genome database. Among the 61,486 unrelated individuals sequenced as part of various disease-specific and population genetic studies available at the Exome Aggregation Consortium (ExAC) website (accessed January 05, 2015), the g.57475100G > T allele was observed in 104 out of 122778 allele counts but of those, only 24 are reported as hemi/homozygous. Of note, this dataset includes cohorts of patients with mental illnesses. The variant is predicted damaging via all tested prediction tools, including SIFT [34] (score 0.041), PROVEAN ([35]; score −2.85) and Polyphen2 ([36]; score 0.998) software systems; according to the Combined Annotation Dependent Depletion (CADD) [37] scoring system, the variant has a Phred-score of 16.93, considered as likely functionally impactful. X-inactivation studies showed random X-inactivation in the mother.

**Figure 2:**
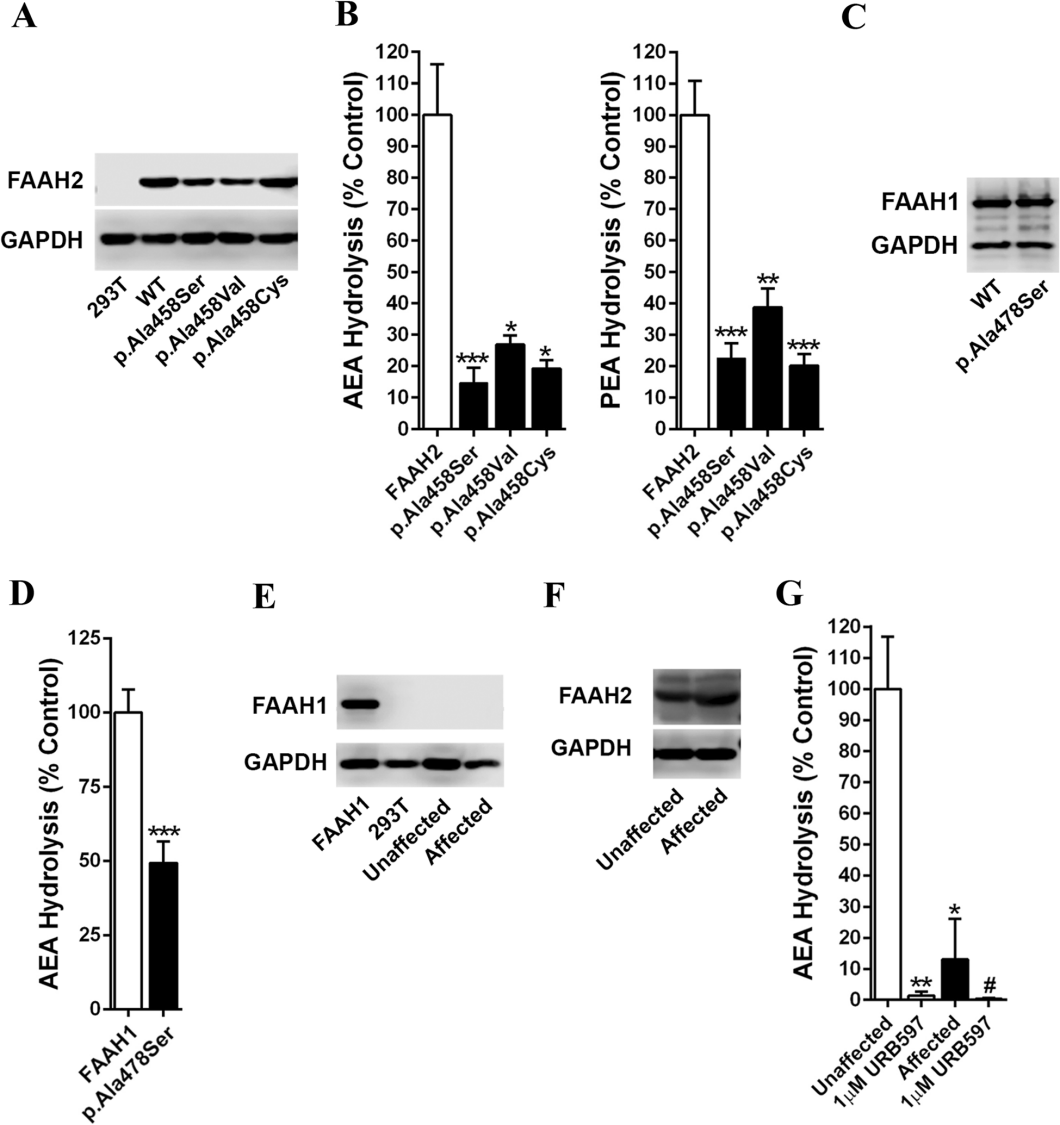
**Functional analysis of** ***FAAH2*** **mutations. (A)** Expression of FLAG-tagged FAAH2 and FAAH2 mutants in 293T cells. The blots were probed with anti-FLAG (Sigma # F1804) and anti-glyceraldehyde 3-phosphate dehydrogenase (GAPDH, Abcam #ab8245) antibodies. **(B)** AEA and PEA hydrolysis by cell homogenates expressing FAAH2 and FAAH2 mutants. Results were analyzed using one-way ANOVA followed by Dunnett’s post hoc analysis. *, p < 0.05; **, p < 0.01; ***, p < 0.001 (n = 5–7). **(C)** Expression of FLAG-tagged FAAH1 and the FAAH1 p.Ala478Ser mutant in 293T cells. Blots were probed with an anti-FLAG antibody. **(D)** AEA hydrolysis by WT and p.Ala478Ser FAAH1 ***, p < 0.001 (n = 4). **(E)** Human fibroblasts do not express *FAAH1*. Fibroblasts from a control patient and the FAAH2 p.Ala458Ser affected patient were probed with anti-FAAH1 antibodies (Abcam #ab54615). *FAAH1* transfected and untransfected 293T cells served as controls. **(F)** Expression of *FAAH2* in fibroblasts from the affected p.Ala458Ser patient and an unaffected control. Blots were probed with an anti-FAAH2 antibody (Abcam, ab103724). **(G)** AEA hydrolysis by control fibroblasts and affected FAAH2 p.Ala458Ser fibroblasts in the absence and presence of URB597. *, p < 0.05; **, p < 0.01 versus unaffected control. #, p < 0.05 versus affected control (n = 4).

We subsequently generated and expressed the p.Ala458Ser FAAH2 mutant in human 293T cells to explore the functional consequences of the p.Ala458Ser mutation found in our patient. Despite repeated efforts to enhance its expression, the p.Ala458Ser mutant was consistently expressed at a reduced level (~30-40%) compared to the wild type (WT) enzyme (Figure 2A). Enzymatic analysis using two established FAAH2 substrates, AEA and palmitoylethanolamide (PEA), revealed that substrate hydrolysis by the p.Ala458Ser mutant was significantly lower compared with the WT enzyme (Figure 2B). The importance of alanine at p.458 is supported by its high conservation among the amidase signature family of enzymes and absolute conservation among FAAH2 orthologs (Additional file 2: Figures S1 and S2). Alanine at p.458 in FAAH2 corresponds to alanine at p.478 in FAAH1. We confirmed that similar to FAAH2, the p.Ala478Ser FAAH1 mutant possessed reduced catalytic activity (Figure 2C and D) despite similar levels of expression, indicating that the importance of this residue is not confined to FAAH2 and may extend to many members of the amidase signature family.

**Figure 3:**
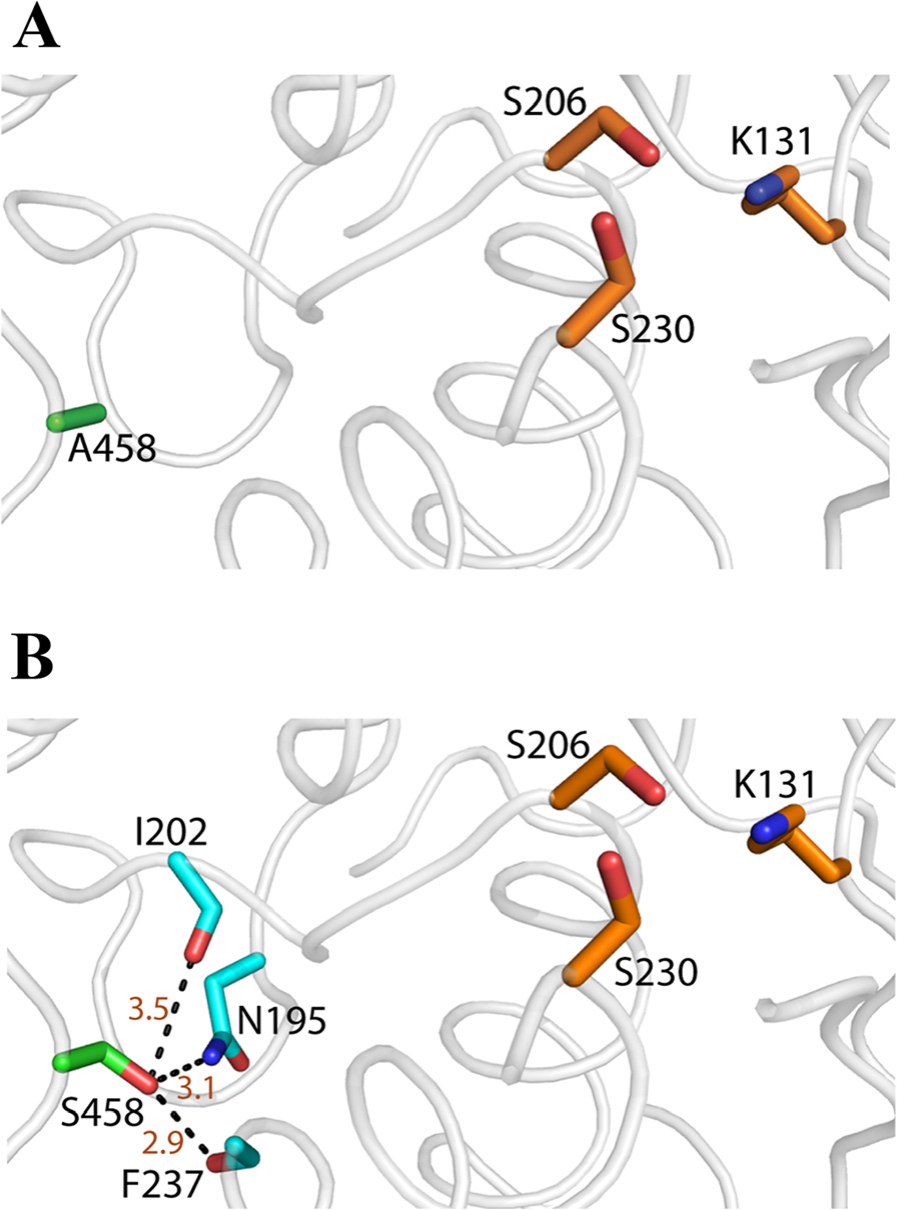
**Modeled structural environments of the disease-causing mutations in FAAH2.** Modeled structure of human FAAH2. The protein backbone is shown as a cartoon (white) with the side chains of A458 (green) and the catalytic triad residues (K131, S206, S230; orange) shown as sticks. **(B)** Additional interactions formed by the p.Ala458Ser mutation. Potential hydrogen bonds are observed between the side chain of Ser458 (green sticks) and the side chain of N195 or the carbonyl oxygens of I202 and F237 (cyan sticks). Distances in angstroms between interacting atoms are shown in dark red.

Alanine at p.458 is situated in a region that is in close proximity to a loop region that houses p.Ser206, one of the two active site serines in this enzyme class ([38]; Figure 3A). Molecular modeling suggests that substitution of this residue with a serine may result in additional hydrogen bonding interactions with surrounding residues (e.g., p.Asn195) (Figure 3B). To examine the importance of potential hydrogen bonding interactions induced by the serine mutation, we mutated alanine at p.458 to cysteine or valine, residues that possess reduced or no hydrogen bonding potential, respectively. Surprisingly, despite robust expression, the p.Ala458Cys FAAH2 mutant displayed catalytic activity that was similar in magnitude to the p.Ala458Ser mutation (Figure 2A and B). Furthermore, the p.Ala458Val mutant was expressed at a similar level as the p.Ala458Ser mutant but possessed only marginally higher catalytic activity, suggesting that substitution of alanine at p.458 with bulkier amino acids, rather than additional hydrogen bonding potential, may account for the defects in FAAH2 activity in the mutants. Collectively, our data establish alanine at p.458 as a residue that is essential for substrate hydrolysis by FAAH2 and its importance is highlighted by its high degree of conservation among amidase signature enzymes.

To explore effects of the *FAAH2* mutation in patient tissues, we examined AEA hydrolysis in cultured fibroblasts derived from our patient and a corresponding unaffected control. Importantly, human fibroblasts do not express *FAAH1* (Figure 2E) but robustly express *FAAH2* (Figure 2F), thus permitting an examination of this *FAAH2* mutation upon endocannabinoid metabolism. AEA hydrolysis by fibroblasts bearing the p.Ala458Ser mutation was significantly lower compared to unaffected control fibroblasts (Figure 2G). Treatment of fibroblasts with URB597, a selective dual FAAH1 and FAAH2 inhibitor [21,39], completely abolished AEA hydrolysis, thereby confirming that AEA hydrolysis observed in fibroblasts was mediated by FAAH2 (Figure 2G).

To further substantiate the functional consequences of the p.Ala458Ser mutation, we profiled the levels of the endocannabinoids, AEA and 2-AG, and related N-acylethanolamines, PEA and oleoylethanolamide (OEA), in fibroblasts from our patient and two unrelated controls shown in Additional file 2: Figure S3. Our results reveal an elevation of AEA levels in the affected fibroblasts compared to the controls and provide further support of defective AEA hydrolysis by the FAAH2 p.Ala458Ser mutant. Furthermore, detailed lipidomic analysis on serum from our patient showed perturbations in multiple lipid species, with elevations in many long chain species, most marked with lyso-phosphatidyl choline as shown in Additional file 3: Table S2.

## Discussion

We have demonstrated for the first time functional impairments in FAAH2 activity in a patient with neurologic and psychiatric symptoms who has a missense mutation in *FAAH2* that results in reduced enzyme activity. Several lines of evidence point to a possible causal relationship between defects in FAAH2 activity and the psychiatric/neurologic phenotype in this patient. Reduced activity of FAAH1 has been associated with other psychiatric illnesses [20] so it is reasonable to hypothesize that alterations in the ECS caused by changes in FAAH2 activity could also have psychiatric consequences. Our patient has a phenotype dominated by anxiety symptoms and alterations in the ECS have been shown in many psychiatric conditions including anxiety for which a biphasic response (low levels of ECS activation reducing anxiety and higher levels of activation increasing anxiety) has been demonstrated [6]. Our patient has some mild specific learning disabilities and *FAAH1* knock-out mice have been shown to exhibit learning deficits that can be reversed with the cannabinoid receptor antagonist rimonabant [40]. Our patient had early onset of autistic features and several lines of evidence link alterations in the ECS to autistic symptoms [16]. Neuroligin-3 mutations associated with autism disrupt endocannabinoid signaling [15]. *FAAH1* knockout mice also have a behavioral phenotype characterized as reduced emotionality [41]. It must be acknowledged however that mice lack *FAAH2* [21] and that FAAH1 and FAAH2 have different substrate affinities [21], so any comparison of the human FAAH2 deficiency phenotype to that of the *FAAH1* knock-out mouse must be interpreted with caution.

Our patient had a vertical supranuclear gaze palsy which we postulate could be related to reduced FAAH2 activity within the intracellular lipid droplets that serve as sites of cholesterol ester storage and mobilization [22]. *FAAH2* is expressed in a number of human tissues [4]. Although the primary function of FAAH2 in these tissues remains unclear, lipid droplet localization is required for active hydrolysis of endocannabinoids by FAAH2 [22]. Lipid droplets are an important site of triacylglyeride and cholesteryl-ester storage and trafficking and endocannabinoid metabolism by FAAH2 may influence these pathways. Intracellular trafficking of cholesterol and some fatty acids occurs through a pathway involving the Niemann Pick C1 transporter and modulation of its function alters the eicosanoid pool in cells [42]. In Niemann Pick C, there is defective intracellular lipid trafficking and patients with this condition develop vertical supranuclear gaze palsy over time [43]. Therefore, we hypothesize that a reduction of FAAH2 activity as a result of the mutation in our patient could alter the levels of FAAH2 substrates and products and feedback to alter cholesterol trafficking at the level of the lipid droplet and lead to this unusual clinical feature.

The persistent elevations of medium chain acylcarnitines in both serum and bloodspot in our patient were suggestive of a dysregulation of fatty acid oxidation (FAO). The pattern was not suggestive of an enzyme-specific block in beta-oxidation but rather was reminiscent of the pattern seen secondary to medium chain triglyceride supplementation, with elevations resulting from increased flux through the pathway. The role of endocannabinoids in FAO is complex with both agonistic and antagonistic effects depending on the receptor system and tissue involved. In muscle cells, agonists of the type 2 cannabinoid receptor have been shown to promote FAO through transcriptional upregulation of carnitine palmitoyltransferase I [44]. Such an effect would be consistent with the acylcarnitine pattern seen in our patient (increased flux).

We are aware of 2 other citations where *FAAH2* mutations have been identified in human patients with neurologic diseases. In a study of humans with autism spectrum disorder, 2 males with validated nonsense mutations in *FAAH2* are listed in a supplementary appendix [27]. In a study of patients with X-linked intellectual disability [26], two brothers were identified with a deletion involving *FAAH2*. Clinical and biochemical details of the patients were not provided in either citation (and efforts to obtain such information were not successful); furthermore, the impact of the mutations on enzymatic activity were not quantified. The p.Ala458Ser mutation was found in 104 alleles in the Exome Aggregation Consortium website (accessed January 5 2015) but, as the pedigree of our patient suggests an X-linked recessive pattern of inheritance, only the 24 male alleles carrying this mutation are relevant to our case. The fact that this mutation has been identified in other patients in this large data set is not surprising because the Exome Aggregation Consortium includes patients with mental illnesses. If *FAAH2* mutations are indeed related to common conditions like autism spectrum disorder and mental illness, then it could be expected that similar mutations could be identified in multiple patients. Further research however is required to prove a causal relationship between *FAAH2* mutations and these common conditions.

Our hypothesis that the functional impairments in FAAH2 activity could contribute to neurological symptoms in humans has limitations. Information is conflicting on the distribution of FAAH2 in human brain. Some reports (www.brainspan.org) but not others [21] have been able to identify *FAAH2* transcripts in human brain. Much of the information supporting this hypothesis has been derived from *FAAH1* knockout mice and, as mice lack *FAAH2* [21] and FAAH1 and FAAH2 have different substrate affinities [21], the effects of the single gene knockout in mice may not be applicable to humans. Clinical details from other patients with mutations in *FAAH2* are needed to see if the unusual clinical features seen in our patient (supranuclear gaze palsy, abnormal acylcarnitine profile) are a consistent feature of the phenotype.

## Conclusions

This is the first comprehensive clinical and biochemical report of FAAH2 deficiency in humans. We propose that the neuropsychiatric features may result from FAAH2 deficiency and identification and characterization of more cases are required to delineate the full spectrum and to further confirm the demonstrated relation between symptom severity and the degree of enzymatic impairment. Areas deserving further research include FAAH2 distribution and ECS actions in human brain (www.brainspan.org) as well as the precise functions of FAAH2 substrates –both known and unknown. Finally, *FAAH2* mutations should be considered in the differential diagnosis of patients with undiagnosed neuropsychiatric impairment.

## Additional files

Additional file 1: Table S1. Variants identified on whole exome sequencing in our male patient with neurologic and psychiatric features. Additional file 2: Figure S1. Sequence comparison of selected amidase signature family members. The sequences of Bacillus subtilis Glu-tRNAGln amidotransferase subunit A, Pseudomonas putida mandelamide hydrolase, Homo sapiens *FAAH2*, Homo sapiens *FAAH1*, and Talaromyces marneffei PM1 Acetamidase are shown. Asterisk denotes the location of p.Ala458 in FAAH2. The sequences were aligned with Clustal omega and depicted with boxshade. **Figure S2.** Sequence alignment of FAAH2 orthologs. Shown are the sequences of *FAAH2* from Bactrocera dorsalis, Manacus vitellinus, Chinchilla lanigera, Callithrix jacchus, Macaca mulatta, Homo sapiens, Pongo abelii, Pteropus Alecto, and Myotis lucifugus. Asterisk denotes the location of p.Ala458. **Figure S3.** Endocannabinoid and N-acylethanolamine levels in fibroblasts. PEA, OEA, AEA, and 2-AG levels were quantified in fibroblasts derived from the FAAH2 p.Ala458Ser patient and two unaffected controls. Quantification was performed as described previously (41). AEA – anandamide, 2-AG - 2-arachidonoylglycerol (2-AG), PEA - palmitoylethanolamide, OEA - oleoylethanolamide. Additional file 3: Table S2. Lipidomic studies from serum of our patient.

## Abbreviations

ECS: Endocannabinoid system
AEA: Anandamide
FAAH: Fatty Acid Amide Hydrolase
MAF: Minor allele frequency
MADD: Multiple acylCoA dehydrogenase deficiency
MRM: Multiple reaction monitoring
CADD: Combined Annotation Dependent Depletion
WT: Wild Type
FAO: Fatty Acid Oxidation
