# Supplementary material for: Defects in fatty acid amide hydrolase 2 in a male with neurologic and psychiatric symptoms
Source: Orphanet J Rare Dis. 2015 Mar 28;10:38. doi: 10.1186/s13023-015-0248-3 (PMC4423390; doi:10.1186/s13023-015-0248-3)
Supplement: Additional file 1: Table S1. — Variants identified on whole exome sequencing in our male patient with neurologic and psychiatric features. [file 13023_2015_248_MOESM1_ESM.pdf]

Table S1. Variants identified on whole exome sequencing in our male patient with neurologic and psychiatric features

Gene symbol	Gene description	Chr	Coordinate	Reference	Alternative	Mutation Impact	Mutation damage	Gene function
**Hemizygous model**								
FAAH2	FAAH2:fatty acid amide hydrolase 2	chrX	57475100	G	T	missense_variant	p.Ala458Ser/c.1372G>T	Endocannabanoid metabolism; see report
KIAA1210	KIAA1210:KIAA1210	chrX	118230653	C	T	missense_variant	p.Ser357Asn/c.1070G>A	Unknown but protein coding
KIAA1210	KIAA1210:KIAA1210	chrX	118239010	G	A	missense_variant	p.Ser338Leu/c.1013C>T	Unknown but protein coding
AKAP14	AKAP14:A kinase (PRKA) anchor protein 14	chrX	119054496	A	G	missense_variant	p.Asn114Asp/c.340A>G	Anchors protein kinase A in cilia
TXLNG	TXLNG:taxilin gamma	chrX	16846277	A	G	missense_variant	p.Ile55Val/c.163A>G	Binds to syntaxin and may play a role in cell cycle regulation
ZMYM3	ZMYM3:zinc finger, MYM-type 3	chrX	70469939	C	G	missense_variant	p.Arg300Pro/c.899G>C	Part of histone deacetylase containing protein complex
NRK	NRK:Nik related kinase	chrX	105167411	A	G	missense_variant	p.Asp971Gly/c.2912A>G	Actin polymerization in embryogenesis
Compound heterozygous model								
MUC16	MUC16:mucin 16, cell surface associated	chr19	9069507	G	T	missense_variant	p.Thr5980Asn/c.17939C>A	Cell surface associated mucin
MUC16	MUC16:mucin 16, cell surface associated	chr19	9050111	G	T	missense_variant	p.Thr10507Asn/c.31520C>A	
CFTR	CFTR:cystic fibrosis transmembrane conductance regulator (ATP-binding cassette sub-family C, member 7)	chr7	117171029	G	A	missense_variant	p.Arg36His/c.107G>A	Chloride channel
CFTR	CFTR:cystic fibrosis transmembrane conductance regulator (ATP-binding cassette sub-family C, member	chr7	117199644	ATCT	A	disruptive_inframe_deletion	p.Ile477_Phe478del/c.1430_1432delTCT	

	7)							
TTN	TTN:titin	chr2	179472223	A	G	missense_variant	p.Ile8666Thr/c.25997T>C	Striated muscle protein
TTN	TTN:titin	chr2	179395554	GC	AA	missense_variant	p.Ala32695Phe/c.98083GC>TT	
KEAP1	KEAP1:kelch-like ECH-associated protein 1	chr19	10597342	TCTG	T	inframe_deletion	p.Gln620del/c.1858_1860delCAG	Targets proteins for ubiquination
KEAP1	KEAP1:kelch-like ECH-associated protein 1	chr19	10597392	A	G	missense_variant	p.Val604Ala/c.1811T>C	
HIVEP2	HIVEP2:human immunodeficiency virus type I enhancer binding protein 2	chr6	143095682	C	A	missense_variant	p.Gly65Val/c.194G>T	Binds to viral promoters; may be involved in T-cell activation
HIVEP2	HIVEP2:human immunodeficiency virus type I enhancer binding protein 2	chr6	143094843	T	C	missense_variant	p.Ile345Val/c.1033A>G	
De novo mutation								
MYO1H	MYO1H:myosin IH	chr12	109845660	T	A	missense_variant	p.Val350Asp/c.1049T>A	Myosin
