# Supplementary figures and images for: Defects in fatty acid amide hydrolase 2 in a male with neurologic and psychiatric symptoms
Source: Orphanet J Rare Dis. 2015 Mar 28;10:38. doi: 10.1186/s13023-015-0248-3 (PMC4423390; doi:10.1186/s13023-015-0248-3)

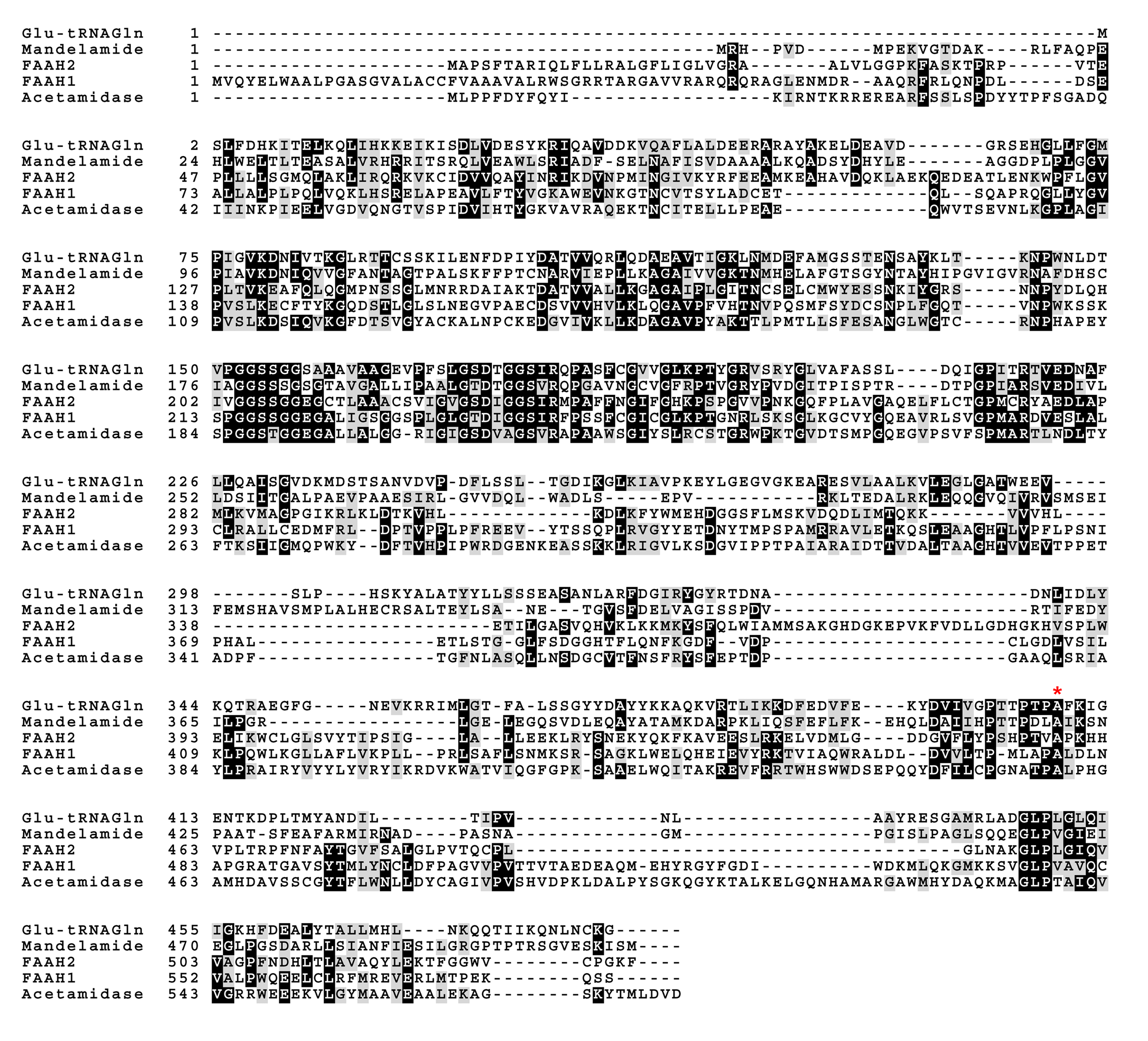

Supplement: Additional file 2: Figure S1. — Sequence comparison of selected amidase signature family members. The sequences of Bacillus subtilis Glu-tRNAGln amidotransferase subunit A, Pseudomonas putida mandelamide hydrolase, Homo sapiens FAAH2, Homo sapiens FAAH1, and Talaromyces marneffei PM1 Acetamidase are shown. Asterisk denotes the location of p.Ala458 in FAAH2. The sequences were aligned with Clustal omega and depicted with boxshade. Figure S2. Sequence alignment of FAAH2 orthologs. Shown are the sequences of FAAH2 from Bactrocera dorsalis, Manacus vitellinus, Chinchilla lanigera, Callithrix jacchus, Macaca mulatta, Homo sapiens, Pongo abelii, Pteropus Alecto, and Myotis lucifugus. Asterisk denotes the location of p.Ala458. Figure S3. Endocannabinoid and N-acylethanolamine levels in fibroblasts. PEA, OEA, AEA, and 2-AG levels were quantified in fibroblasts derived from the FAAH2 p.Ala458Ser patient and two unaffected controls. Quantification was performed as described previously (41). AEA – anandamide, 2-AG - 2-arachidonoylglycerol (2-AG), PEA - palmitoylethanolamide, OEA - oleoylethanolamide. [file 13023_2015_248_MOESM2_ESM.zip › 13023_2015_248_FigS1.tiff]

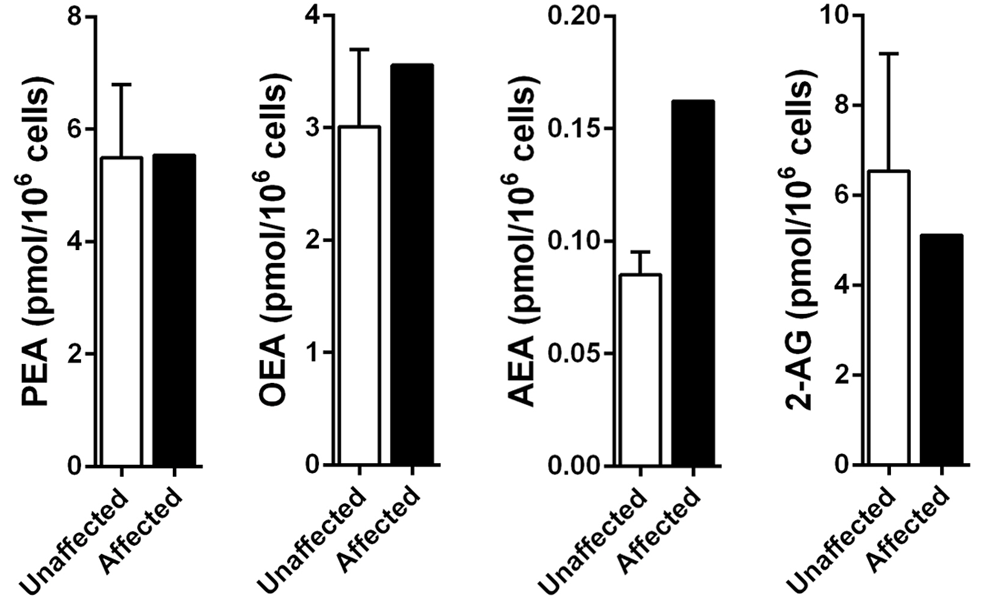

Supplement: Additional file 2: Figure S1. — Sequence comparison of selected amidase signature family members. The sequences of Bacillus subtilis Glu-tRNAGln amidotransferase subunit A, Pseudomonas putida mandelamide hydrolase, Homo sapiens FAAH2, Homo sapiens FAAH1, and Talaromyces marneffei PM1 Acetamidase are shown. Asterisk denotes the location of p.Ala458 in FAAH2. The sequences were aligned with Clustal omega and depicted with boxshade. Figure S2. Sequence alignment of FAAH2 orthologs. Shown are the sequences of FAAH2 from Bactrocera dorsalis, Manacus vitellinus, Chinchilla lanigera, Callithrix jacchus, Macaca mulatta, Homo sapiens, Pongo abelii, Pteropus Alecto, and Myotis lucifugus. Asterisk denotes the location of p.Ala458. Figure S3. Endocannabinoid and N-acylethanolamine levels in fibroblasts. PEA, OEA, AEA, and 2-AG levels were quantified in fibroblasts derived from the FAAH2 p.Ala458Ser patient and two unaffected controls. Quantification was performed as described previously (41). AEA – anandamide, 2-AG - 2-arachidonoylglycerol (2-AG), PEA - palmitoylethanolamide, OEA - oleoylethanolamide. [file 13023_2015_248_MOESM2_ESM.zip › 13023_2015_248_FigS3.tiff]

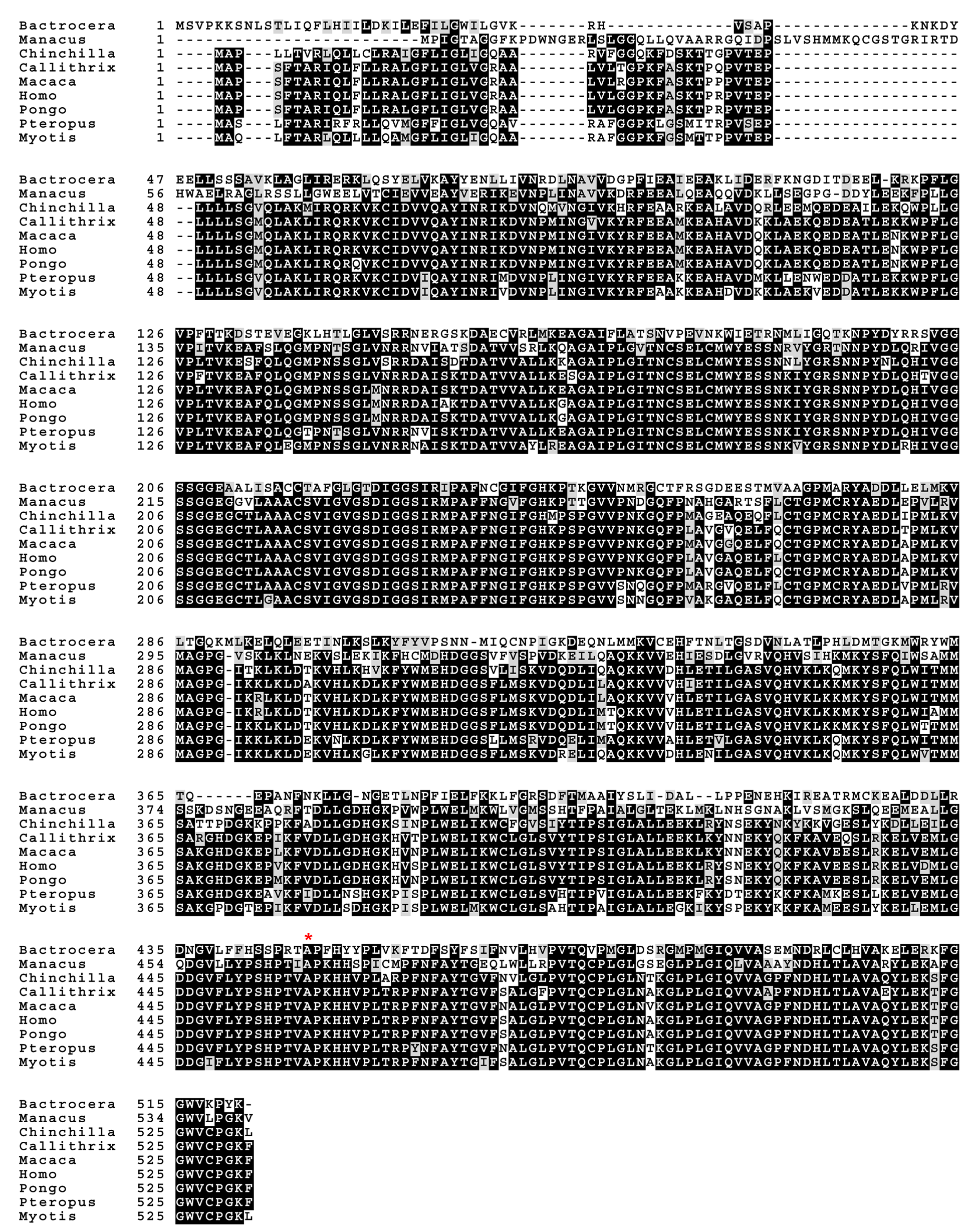

Supplement: Additional file 2: Figure S1. — Sequence comparison of selected amidase signature family members. The sequences of Bacillus subtilis Glu-tRNAGln amidotransferase subunit A, Pseudomonas putida mandelamide hydrolase, Homo sapiens FAAH2, Homo sapiens FAAH1, and Talaromyces marneffei PM1 Acetamidase are shown. Asterisk denotes the location of p.Ala458 in FAAH2. The sequences were aligned with Clustal omega and depicted with boxshade. Figure S2. Sequence alignment of FAAH2 orthologs. Shown are the sequences of FAAH2 from Bactrocera dorsalis, Manacus vitellinus, Chinchilla lanigera, Callithrix jacchus, Macaca mulatta, Homo sapiens, Pongo abelii, Pteropus Alecto, and Myotis lucifugus. Asterisk denotes the location of p.Ala458. Figure S3. Endocannabinoid and N-acylethanolamine levels in fibroblasts. PEA, OEA, AEA, and 2-AG levels were quantified in fibroblasts derived from the FAAH2 p.Ala458Ser patient and two unaffected controls. Quantification was performed as described previously (41). AEA – anandamide, 2-AG - 2-arachidonoylglycerol (2-AG), PEA - palmitoylethanolamide, OEA - oleoylethanolamide. [file 13023_2015_248_MOESM2_ESM.zip › 13023_2015_248_FigS2.tif]
