# Supplementary material for: Defects in fatty acid amide hydrolase 2 in a male with neurologic and psychiatric symptoms
Source: Orphanet J Rare Dis. 2015 Mar 28;10:38. doi: 10.1186/s13023-015-0248-3 (PMC4423390; doi:10.1186/s13023-015-0248-3)
Supplement: Additional file 3: Table S2. — Lipidomic studies from serum of our patient. [file 13023_2015_248_MOESM3_ESM.doc]

| Metabolites | Patient 1 | CONTROL | p-value  ( after Bonferroni correction) | CASE/ CRONTROL |
| --- | --- | --- | --- | --- |
| Number of cases | 1 | 10 | - |  |
| C3:1 | 0.057 | 0.050±0.002 | <0.001** | Up |
| C18:2 | 0.129 | 0.068±0.028 | <0.001** | Up |
| lysoPC a C16:0 | 285.427 | 170.815±32.855 | <0.001** | Up |
| lysoPC a C17:0 | 5.603 | 3.108±0.799 | <0.001** | Up |
| lysoPC a C18:0 | 84.011 | 48.337±12.368 | <0.001** | Up |
| lysoPC a C18:2 | 55.782 | 38.179±6.019 | <0.001** | Up |
| lysoPC a C20:3 | 5.541 | 2.654±0.723 | <0.001** | Up |
| PC aa C38:1 | 0.409 | 1.705±0.441 | <0.001** | Down |

**Table S2. Lipidomic studies from serum of our patient.**

The plasma sample from our patient was tested against 10 control samples to analyze levels of 120 metabolites. We have applied a targeted quantitative metabolomics approach to analyze the serum samples using a combination of direct injection mass spectrometry (Absolute*IDQ*™ Kit) with a reverse-phase LC-MS/MS Kit as per the method cited below. Because of a single sample disease case, the one sample t-test was used to examine the difference of metabolic phenotypes between disease and control. The Bonferroni corrected significance level of p = 0.05/120 = 0.0004 was used to identify levels of metabolites that were significantly different in Patient 1 versus control samples. Only values reached statistical significance are shown. C3:1 (Propenoylcarnitine); C18:2 (Octadecadienylcarnitine); lysoPC a C16:0 (lysoPhosphatidylcholine acyl C16:0); lysoPC a C17:0 (lysoPhosphatidylcholine acyl C17:0); lysoPC a C18:0 (lysoPhosphatidylcholine acyl C18:0); lysoPC a C18:2 (lysoPhosphatidylcholine acyl C18:2); lysoPC a C20:3 (lysoPhosphatidylcholine acyl C20:3); PC aa C38:1 (Phosphatidylcholine diacyl C38:1)

Reference:

Walsh, B.H., Broadhurst, D.I., Mandal, R., Wishart, D.S., Boylan, G.B., Kenny, L.C., 11 and Murray, D.M. (2012) The metabolomic profile of umbilical cord blood in neonatal 12 hypoxic ischaemic encephalopathy. PLOS One., 7, e50520.
